# Early and Moderate Sensory Stimulation Exerts a Protective Effect on Perilesion Representations of Somatosensory Cortex after Focal Ischemic Damage

**DOI:** 10.1371/journal.pone.0099767

**Published:** 2014-06-10

**Authors:** Christian Xerri, Yoh'i Zennou-Azogui

**Affiliations:** Neurosciences Intégratives et Adaptatives, Aix-Marseille Université, Centre National de la Recherche Scientifique, Unité Mixte de Recherche 7260, Fédération de Recherches Comportement-Cerveau-Cognition 3512, Marseille, France; University of South Florida, United States of America

## Abstract

Previous studies have shown that intensive training within an early critical time window after focal cortical ischemia increases the area of damaged tissue and is detrimental to behavioral recovery. We postulated that moderate stimulation initiated soon after the lesion could have protective effects on peri-infarct cortical somatotopic representations. Therefore, we have assessed the effects of mild cutaneous stimulation delivered in an attention-demanding behavioral context on the functional organization of the perilesion somatosensory cortex using high-density electrophysiological mapping. We compared the effects of 6-day training initiated on the 3rd day postlesion (early training; ET) to those of same-duration training started on the 8th day (delayed training; DT). Our findings confirm previous work showing that the absence of training aggravates representational loss in the perilesion zone. In addition, ET was found to be sufficient to limit expansion of the ischemic lesion and reduce tissue loss, and substantially maintain the neuronal responsiveness to tactile stimulation, thereby preserving somatotopic map arrangement in the peri-infarct cortical territories. By contrast, DT did not prevent tissue loss and only partially reinstated lost representations in a use-dependent manner within the spared peri-infarct cortical area. This study differentiates the effects of early versus delayed training on perilesion tissue and cortical map reorganization, and underscores the neuroprotective influence of mild rehabilitative stimulation on neuronal response properties in the peri-infarct cortex during an early critical period.

## Introduction

Following stroke, rehabilitative procedures are aimed at strengthening neuroplasticity mechanisms that subserve functional recovery. Recruitment of multiple regions in both ipsi- and contra-lesional hemispheres represents a vicariation process operating temporarily or permanently, depending on the lesion size and behavioral experience. There is accumulating evidence that restoration of lost function is largely dependent upon the cortex adjacent to the infarct, which emphasizes the importance of preserving the perilesion penumbral area, a cortical zone of reversible damage [see 1 and 2, for reviews]. The axonal and dendritic circuitry of neurons located 300 micrometers outside an ischemic zone in mice can be relatively undamaged, suggesting that they may crucially contribute to functional recovery [3]. Moreover, the peri-infarct tissue that exhibits substantial changes in neuronal excitability, dendritic spines and synaptic connections [4], [5] has a strong potential for remapping sensory, motor and cognitive functions. Any intervention preserving the molecular and cellular mechanisms of plasticity within the surround of ischemic lesions may therefore facilitate later adaptive cortical changes and promote functional recovery.

Despite remarkable advances in our understanding of sensory and motor map remodeling in peri-infarct cortex, important questions need to be resolved regarding the time window and intensity of practice to optimize rehabilitative intervention in view of the temporal constraints on cortical plasticity and the uncertainty regarding the beneficial effects of early exercise following stroke. Early rehabilitative intervention seems to be more effective in restoring the impaired functions as it tends to prevent learned non-use [6] or compensatory behavioral strategies impeding restoration [7]. However, rehabilitative practice following ischemic damage remains a challenging issue, as the perilesion tissue is particularly vulnerable to behavioral experience. It has been shown that early rehabilitative practice may have beneficial or adverse effects depending on its time of initiation and intensity. Interestingly, 120 min of whisker stimulation was found to exacerbate stroke damage if initiated 3 hours after the lesion, whereas a completely protective effect was observed when stimulation was delivered within 2 hours of ischemic onset [8]. Overuse of the impaired forelimb through intact forelimb immobilization (constraint-induced movement therapy; CMIT) 24 h/day during the first week after injury to the motor cortex was found to aggravate cellular death within the ischemic forelimb zone and compromise functional recovery, whereas forced use during the second week postlesion had no effect on lesion size, but nevertheless impaired recovery [9], [10], [11]. By contrast, moderate-intensity CIMT applied for 8 h/day, from the first day postlesion and during 4 weeks, partially preserved somatosensory-evoked potentials and promoted recovery of forelimb use [12]. Along the same lines, moderate sensorimotor stimulation through housing in enriched environment (EE) 24 to 30 hours after stroke significantly improved outcome without altering infarct volume [13], [14]. The effects of EE combined with rehabilitative training on both neuromorphological changes in the motor cortex of the intact hemisphere and on functional outcome were greater 5 days after stroke, but gradually declined when initiated at 14 or 30 days postlesion [15]. The potential impact of early intervention is highlighted by the macroscopic redistribution of sensory-evoked subthreshold depolarization that may occur in the peri-infarct cortex within an hour after targeted stroke to the forelimb somatosensory area [16]. The rapid redistribution of residual function after stroke is likely to determine the neural circuits that will be sensitive to use-dependent remapping over the following days or weeks [17], [18]. It thus appears that the first days post-injury represent a critical period of cortical tissue vulnerability to sensorimotor stimulation. This early time-window represents an opportunity for rehabilitative intervention, but care must be taken to avoid detrimental effects.

In an earlier study, we showed that a focal ischemic lesion to the S1 forepaw area in rats expands rapidly over the first hours postlesion and results in a degradation of the somatosensory representational zones within the cortical sectors of the forepaw map initially spared by the lesion [19]. The somatotopic map arrangement of cutaneous representations were relatively well preserved in the perilesional zones in the animals exposed to an EE for three weeks after the lesion, i.e. an environment in which mild sensory stimulation was generated through object exploration and social interaction with congeners [20]. By contrast, rats exposed to an impoverished environment exhibited dramatically deteriorated cutaneous maps relative to the initial post-injury maps. However, the specificity of protective effects of behaviorally-driven sensory stimulation could not be assessed in those EE rats. In addition, these findings raise the question as to whether and under what conditions sensory stimulation initiated during the acute stage of ischemia may have beneficial effects.

Little is known about how the experience-dependent plasticity of somatosensory cortical networks in the peri-infarct cortex is influenced by sensory stimulation within the first weeks postlesion. To address this question we used electrophysiological mapping to probe cortical changes with a high spatial resolution. We searched to determine whether early versus delayed mild tactile stimulation differentially impacts the neurons' responses within the vulnerable peri-infarct zone following targeted ischemic injury to the S1 forepaw area. We examined whether perilesion representation zones are preserved or initially disorganized and then remapped in an experience-dependent way under the influence of somatosensory stimulation. Repetitive cutaneous stimulation was delivered during a short-time window, using an attention-demanding and rewarded task, as an attempt to maximize the effects of this rehabilitative intervention despite its short duration.

## Materials and Methods

Experimental procedures were in accordance with the EU directive for the protection of animals (2010/63/EU) and were approved by the Institutional Animal Care and Use Committee of Aix-Marseille University (B 13-055-25). All efforts were made to minimize the number of animals used and their suffering.

### Experimental protocol

Electrophysiological mapping was performed on thirty-two male, 3-month-old adult Long-Evans rats weighing from 300 to 350 g. After weaning (30 days postnatal), rats from different litters were housed for 2 months in groups of three in Plexiglas cages (26.5 cm wide×42.5 cm deep×18 cm high), i.e. under standard laboratory environmental conditions. All animals were maintained on a 12 h∶12 h light∶dark cycle, at constant temperature (23±1°C) with about 60% relative humidity. Food and water were provided ad libitum.

In the present study, we selected right-handed rats using a food-reaching test (see below). All animals were trained to position their right forepaw on a rotating cylinder delivering tactile stimulation (task learning; see below). After we recorded neuronal activity within the cortical area of the right forepaw representation to obtain a control map, we induced a small neurovascular lesion (L) within this area. Recordings were made during the second hour following the lesion induction to assess the initial boundaries of the functionally damaged cortical zone. The animals were returned to the animal care facility and housed in standard environments. They were randomly assigned to postlesion tactile training (T) or no-training (NT) groups. The T group comprised 8 rats subjected to training over the L+3–L+8 postlesion days (early training; ET) and 8 rats trained over the L+8–L+13 period (delayed training; DT). Post-lesion electrophysiological mapping was made on the 9^th^ or 14^ th^ day in ET or DT rats, respectively, to map the spared regions of the forepaw representation. To match the time of mapping between experimental groups, 16 NT animals were separated into two groups, depending on whether postlesion recordings were made at L+9 (NT1) or L+14 (NT2).

### Paw preference determination

We used the modified food-reaching test developed by Tang and Verstynen [21] to assess paw preference in our rats. Briefly, the animals were deprived of food for two days and then individually placed in a narrow Plexiglass chamber located in a metal housing cage with two front openings separated by 1 cm. The openings in the testing cage were small enough to allow access to food pellets (45-mg Precision Food Pellet, Phymep) by a forepaw only, not by the snout. The animal could retrieve the pellets presented in a central position through the left or right opening. An observer scored the numbers of right and left paw reaches. The animals were tested for three days in a row. From the second day on, all rats tended to display stereotypical behavior. The direction of paw preference was measured by a lateralization index computed as (L−R)/(R+L) x 100, where R and L were the number of reaches made by the right and left paws, respectively. An index of +100% and −100% corresponds to a perfect left and right-paw preference. Rats were classified as left-hander or right-hander when the lateralization index reached at least 70% on the third testing day. We found that about 80% of rats were right-handed and nearly 20% of rats were left-handed. Therefore, we retained the right-handed rats in this study.

### Task learning and tactile stimulation training

**Figure 1:**
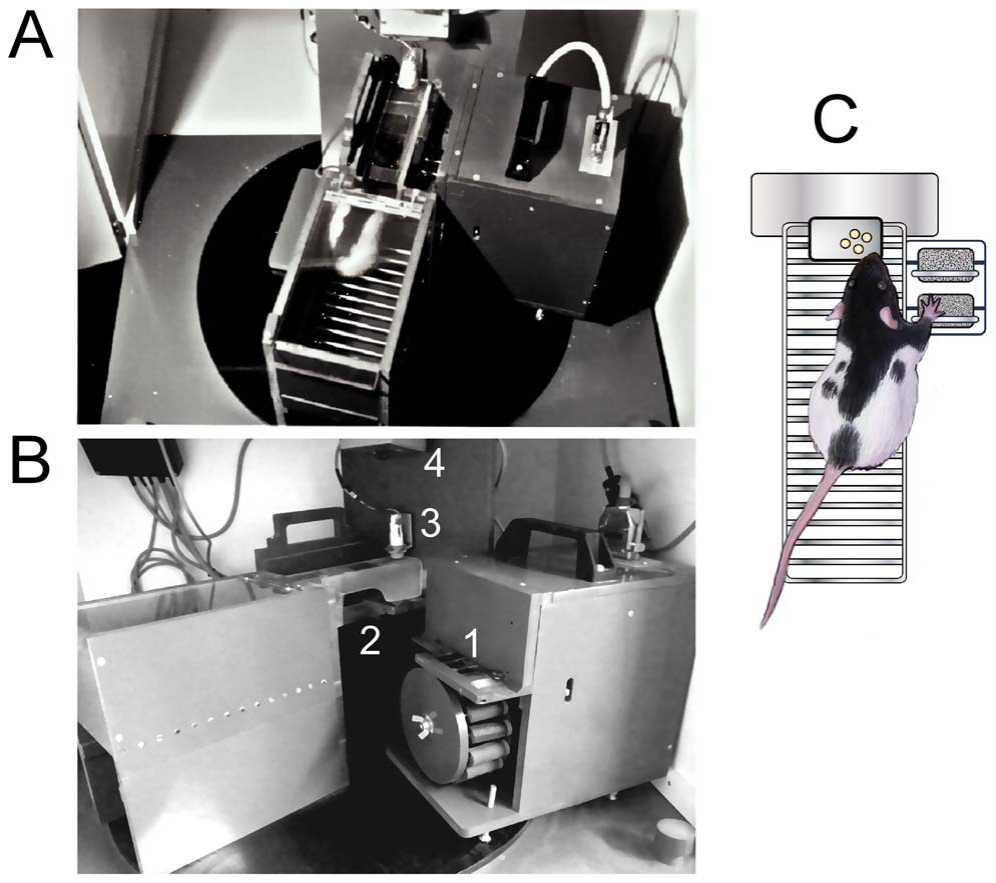
Experimental device used for delivering tactile stimulation. A. Overview of the apparatus in a modified Skinner box located in a sound and light proof container. The rat was trained to stay in a compartment and place its right forepaw on a “tactile barrel”. B. Detailed view of the tactile barrel equipped with rotating cylinders covered with sand paper of different roughnesses. For easier viewing, the block containing the barrel is positioned at 90° from its orientation during training. 1: aperture for paw placement on tactile cylinders; 2: opening allowing the rat to extend its forepaw; 3: food pellet dispenser; 4: infra-red video camera. C. Schematic drawing illustrating the rat's forepaw positioned on a tactile cylinder.

The rats were on a food-restriction schedule before each task learning or training session. Task learning was initiated after two sessions of 1 hour devoted to familiarizing the animals with the stimulation device located in a sound- and light-proof modified Skinner box (Fig. 1). The animals were trained to insert their head and chest in a small rectangular-shaped corridor made of Plexiglass allowing them to extend their right forelimb through an opening. The rats could place their right palm on one of two easily accessible small horizontal bars, so that the glabrous surfaces of digits 2 to 5 were in contact with a rotating cylinder mounted on a barrel and covered with sand paper. The barrel contained 16 cylinders, each covered with sand paper of a different roughness (grit size range: 269-35 microns) (Imétronic, France). Eight different roughnesses were used. The barrel rotation bringing a new cylinder under the rat's forepaw was automatically triggered every 2 min. Barrel and cylinder rotations were in the postero-anterior direction relative to the forepaw. A food tray delivered one food pellet after every period of correct paw placement of 5 sec, as detected by a force sensor (successful attempt). Photoelectric cells detected nose-pokes in the food tray. A software (Imétronic) controlled the pellet distribution, via an electronic interface using signals from the force sensor. A video camera equipped with infrared beams and connected to a TV set allowed the experimenter to monitor the forepaw position on the tactile cylinders. Prelesion task learning was over whenever the rats were able to maintain their forepaw on the stimulation cylinders for five successful attempts in a row, which required 3 to 5 sessions of 30 min each. Given the vulnerability of perilesion tissue to early and intensive sensorimotor experience (see Introduction), cutaneous stimulations during postlesion training were delivered for 6 consecutive days, over 2 daily morning sessions separated by 15 min of rest. The cumulated duration of correct paw placement measured by the dedicated software was set to 30 min per session, so as to provide the animals with the same total duration of tactile stimulation.

### Surgical preparation

Anesthesia was induced with isoflurane short inhalation followed by an intramuscular injection of ketamine (25 mg/kg, Ketalar, Virbac, France) and medetomidine (0.25 mg/kg, Domitor, Orion Pharma, Finland). An analgesic substance, olfenamic acid (10 mg/kg, s.c., Tolfédine, Vetoquinol, France), was injected 20 minutes before surgery. Animals were kept at an areflexive level of anesthesia throughout the experiment by supplemental ketamine-metedomidine combination half doses. The core temperature was continuously monitored by a rectal thermistor probe and maintained constant (38±0.1°C) with a feedback-controlled homeothermic blanket system (Harvard Apparatus Ltd., Kent, UK). The head was held in a stereotactic frame. Surgical and recording procedures were performed under sterile conditions. To prevent cerebral oedema, the posterior neck muscles were resected, and cerebrospinal fluid was drained through an opening in the dura covering the foramen magnum. After a scalp incision and the retraction of attached muscles, SI cortex on the left hemisphere was exposed through a craniotomy (about 16 mm^2^) with bregma as a point of reference (anterior: 2.5 mm; posterior: 1.5 mm; lateral: 2–6 mm). The bone flap was kept in physiological saline at 4°C. The dura protecting the exposed part of the cortex was incised and resected, and the surface of the cortex was bathed in a thin layer of warm silicone fluid (30000 cenistokes; Accumetrics) to prevent drying and oedema formation. At the end of the recording session, after induction of the cortical lesion, the silicone was removed with a wash of warm saline, the exposed cortical area was covered with a piece of sterile Teflon film. The bone flap was reinserted and stabilized with dental acrylic. Connective tissue was closed with absorbable sutures and the scalp with silk sutures. The animal's temperature was monitored until the recovery from anesthesia was complete. Prophylactic administration of antibiotic (Pyocefal, Takeda; 150 mg/kg, i.m., in two daily injections) was administered daily for 7 days. For the postlesion remapping, the anesthesia procedure was repeated. The bone flap was removed to allow access to the cortical zone to be mapped. After completion of the recordings, the animal received a lethal dose of sodium pentobarbital (150 mg/kg, i.p.) and the brain was prepared for histological processing.

### Electrophysiological mapping procedures

**Figure 2:**
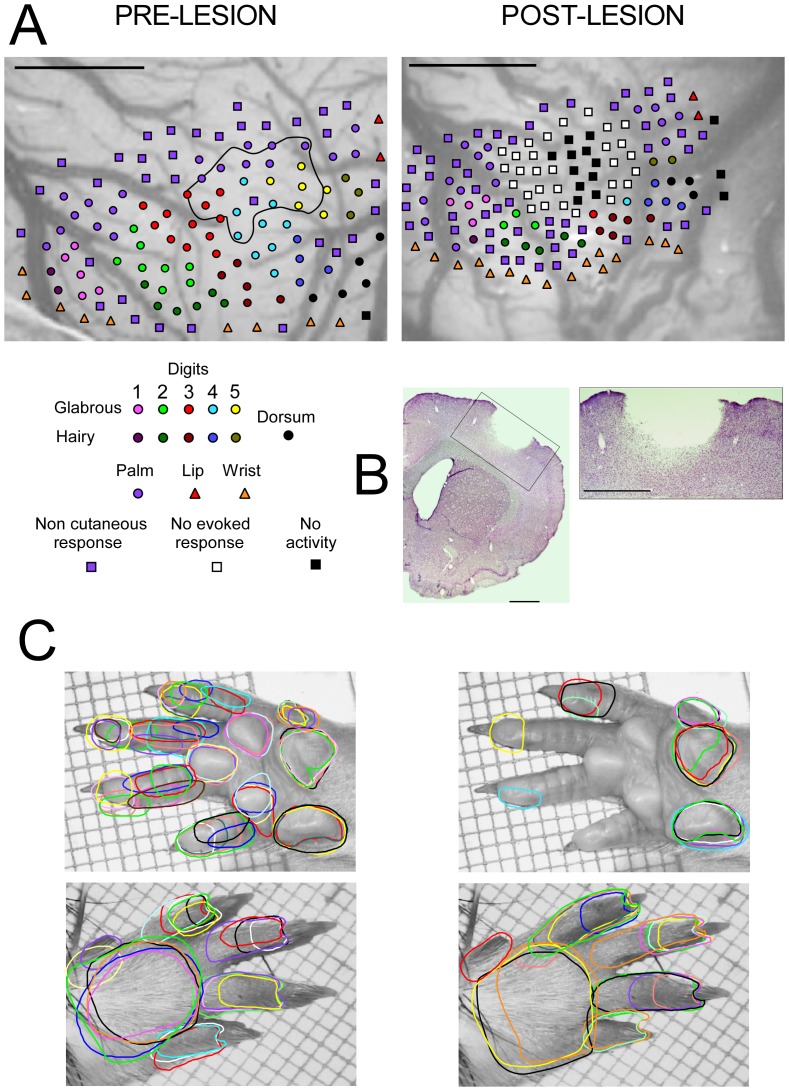
Electrophysiological mapping and cortical lesion location. Map elaboration is based on the response properties of neurons recorded within layer IV of the S1 cortex, before (left) and 9 or 14 days after induction of a focal ischemic lesion (right). **A.** Magnified images of the forepaw cortical area showing recording sites labelled according to whether neuronal responses were induced by just-visible skin indentation (cutaneous) or strong pressure on skin, muscles and joint movements (non-cutaneous) of the contralateral forepaw. Postlesion recording sites displaying spontaneous activity, but no elicited responses, or remaining silent after the lesion induction are also illustrated. The functionally injured area is outlined on the image of intact cortex. **B.** Representative coronal section stained with cresyl violet showing the focal ischemia targeting the forepaw area of S1 (section from a NT rat). The lesion encompassed cortical layers I to V. There was no evidence of damage to subcortical white matter. **C**. Samples of cutaneous receptive fields (RFs) on glabrous and hairy skin surfaces corresponding to the cortical sites illustrated in A.

Magnified images of the exposed parietal cortex, and the ventral and dorsal surfaces of the forepaw contralateral to the cerebral hemisphere to be mapped were digitized by using a high-resolution camera mounted on an operating microscope. Locations of microelectrode penetrations relative to the vasculature of the cortical surface and boundaries of cutaneous receptive fields (RFs) were recorded on the digitized images of cortex and forepaw, respectively, using Map 0.925 software [22]. A parylene-coated tungsten microelectrode (about 1 Megohm at 1 kHz; WPI, UK) was moved perpendicular to the cortical surface in cartesian coordinates by a 3D stepping micromanipulator (Märzhauser, FST; Canada). Using the recording artifact generated by the microelectrode contact with the cortical surface as a zero level, we advanced the electrode to a depth of approximately 650–700 microns, to record responses from small clusters of neurons (two to four) in layer IV [23], [24]. Many recording sites were placed as closely (about 70–80 microns) as possible (Fig. 2 A). Under our recording conditions, the amplitude of the background noise usually ranged from 15 to 20 mV with a signal-to-noise ratio varying from 4 to 6. The multiunit signal was amplified, filtered (bandwidth: 0.5–5 kHz) and displayed on an oscilloscope. This signal was also rectified and passed through a discriminator whose output signal was proportional to the part of the input signal which was higher than an adjustable threshold set just above the background noise. The output of the discriminator was delivered to an audio monitor.

At each recording site, large bursts of activity elicited by natural stimulation allowed us to classify neuronal responses as cutaneous or noncutaneous. Cutaneous RFs were defined as the skin area where just-visible skin indentation or hair deflection elicited reliable changes in multi-unit activity. This stimulation was produced with a fine-tipped, hand-held glass probe and monitored by using magnifying glasses (x4). The ridges running along the glabrous skin of the digits were used as landmarks to delineate the RFs of recorded neurons (Fig. 2 C). Noncutaneous responses were identified by taps and pressure on tendons, intrinsic muscle or joint manipulations while no cutaneous response was found. Cortical sites exhibiting only spontaneous discharges were classified as unresponsive. The thresholds of neuronal responses to skin stimulation were determined using von Frey monofilaments (Stoelting, Semmes-Weinstein aesthesiometer) that apply indenting stimuli at a relatively constant, predetermined force. The most commonly used filaments were 3.22 (diameter: 0.152 mm; bending force: 0.166 g; bending pressure: 9.15 g/mm^2^), 3.61 (0.178 mm; 0.407 g; 16.36 g/mm^2^), and 3.84 (0.203 mm; 0.692 g; 21.39 g/mm^2^). For reproducible measurements, the filaments were used at a relatively constant room temperature (about 24°C). The stimulation consisted in pressing a filament gently against the skin, perpendicular to its surface at the center of the RFs, until the filament began to bend. This procedure was done 5 to 10 times for each filament. We used a stimulus series of increasing and decreasing strengths to determine the mechanical threshold eliciting noticeable changes in neuronal discharge.

After the recording session, we used Canvas software (Deneba) to elaborate maps of the forepaw representation by drawing boundaries around cortical sites whose RFs were restricted to a common forepaw subdivision, i.e. finger or palmar pad. Borders were drawn midway between adjacent recording sites where RFs were located on distinct and separate skin subdivisions. A boundary line crossed cortical sites at which a single RF included different but adjoining skin subdivisions of the forepaw. The same principle was used to draw boundaries encompassing cortical sites where noncutaneous responses were obtained for each forepaw subdivision. Canvas software was further applied to calculate the area of each cutaneous region of the cortical somatosensory map.

### Induction of Cortical Lesion

The silicone fluid was removed and the exposed cortex was bathed in warm physiological saline. A temperature-monitoring electrode (Radionics TCZ; Kopf; USA) was moved perpendicular to the cortex surface by the micromanipulator and was slowly lowered so that its tip (diameter: 0.25 mm) was in contact with the cortical surface. The lesion was performed at about the center of the forepaw map to spare an amount of tissue allowing reorganization of the intact areas surrounding the ischemic zone. This lesion targeted the representational sector of glabrous skin of digits and palm. A 500 MHz radiofrequency current was delivered through the electrode so that the temperature measured at its tip, equipped with a thermistor, was gradually raised to 70°C within 1 min and maintained at this level for 1 min. In addition to destroying neural tissue, the heat generated in the brain tissue induced a focal infarct characterized by a visible occlusion of the vessels, along with a blanching of the cortical zone within the vicinity of the electrode tip. Care was taken to preserve major arteries and veins while occluding their local branches. Radio-frequency-induced hyperthermia produces cellular injury, i.e. coagulation necrosis, neuronal shrinkage, nuclear pyknosis and perineuronal astrocytic swelling [25] that is associated with cerebral ischemia. Moreover, localized hyperthermia increases extracellular glutamate concentration that reaches neurotoxic levels [26].

As in previous studies [27], [28], [29], the functional impact of the lesion was assessed using electrophysiological recordings. The boundaries of the cortical infarct were assessed during the second hour after the lesion induction and drawn midway between adjacent recording sites which displayed normal response characteristics, i.e. spontaneous discharges and clear responses to peripheral stimulation, and those cortical sites which exhibited strongly or totally depressed spontaneous activity and no stimulus-induced responses. The surface area of the lesion was measured on the cortex image using Canvas software.

### Experimental measurements and statistical analysis

**Figure 3:**
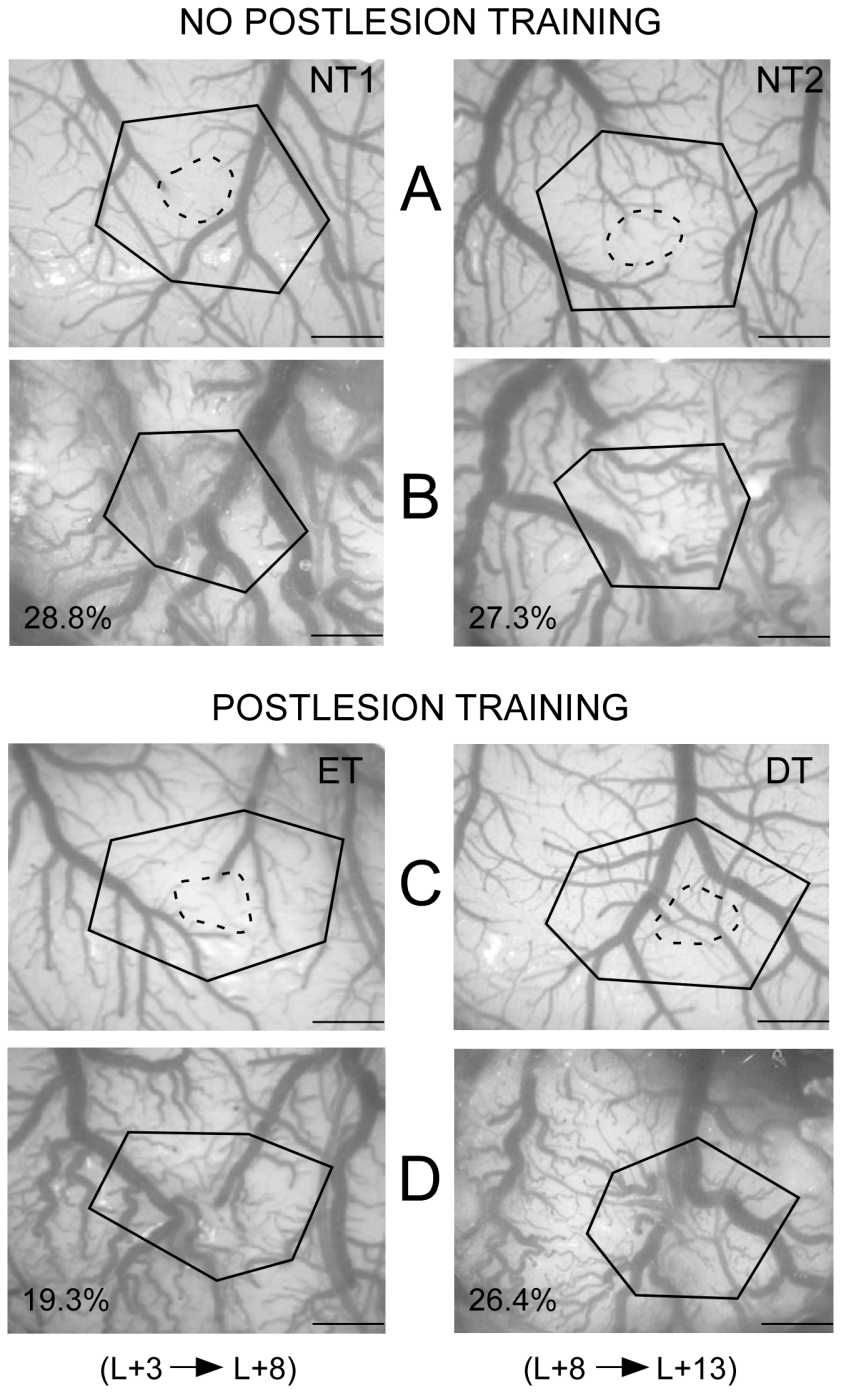
Cortical tissue loss after focal ischemic damage to the S1 forepaw area. Video images of the cortical surfaces taken before (A, C) and 9 days (left column) or 14 days (right column) after the lesion induction (B, D). Images are shown for representative rats not trained (NT1 and NT2) or trained during postlesion days 3 to 8 (ET) or 8 to 13 (DT). The electrophysiologically assessed injured areas are outlined (broken line) on the image of the intact cortex. Polygons based on common vascular landmarks before and after the lesion illustrate the contraction of the cortical area due to tissue loss (percent values). Note that the estimated tissue loss was greater in the NT and DT than in the ET rats.

The assessment of cortical tissue loss was based on the comparison of the area of a prelesion reference polygon with that of a postlesion polygon drawn on cortical images of the forepaw area in S1, using invariant vascular landmarks on the surface of the cortex. The injured zones with the lesion area defined on the basis of neuronal recordings represented about 8 to 10% of the reference polygon surface area and were roughly located at the centers of these polygons. The reference polygon surface areas as well as the electrophysiologically determined injured areas did not differ among rats (see Results). The prelesion vascular landmarks were used to delimit a new polygon on the images of the cortex obtained 9 (NT1 and ET) or 14 days (NT2 and DT) after the lesion (Fig. 3). The areas of the two polygons obtained in each rat were measured and the tissue loss was estimated as a relative decrease in the surface area of the reference polygon. We checked that changing the number and location of reference polygon vertexes, surface area of the reference polygons or exact positioning of the lesions at the centroids of the polygons did not alter our findings. For example, doubling the surface areas of reference polygons, i.e. including larger zones of intact cortex, thus minimizing the relative impact of the lesion, yielded similar differences between groups. Comparison of the shapes of pre- and postlesion polygons in each rat indicates that the contraction due to tissue loss was not isotropic, very likely due to heterogeneity in vascular patterns within a given cortical zone and idiosyncratic differences in this pattern among rats.

For each rat, we calculated the cortical areas serving the main territories of the cutaneous forepaw representation, i.e. those excited by stimulation of digit/palm glabrous or digit/dorsum hairy skin surfaces. We also assessed the areal extent of the injured zone functionally assessed during the second hour following the lesion. The spared portion of the representation was referred to as an “acute” post-lesion area and was estimated by subtracting the injured area from the area of forepaw representation recorded prior to the lesion. The spared area of the representation as measured during the second mapping session was referred to as a chronic post-lesion area. Further loss in the total area of the representation appeared to be the rule. It was expressed as follows: [(chronic area – acute area)/acute area] X 100. This formula was also used to assess more specifically the alteration of representations of digit glabrous skin, palm and digit/dorsum hairy skin as parts of the forepaw representation [20].

The sizes of glabrous and hairy RFs were measured in mm^2^. The RF areas measured in each rat were averaged, then the mean size of the RFs was computed for each group of rats. The stimulation thresholds obtained using the von Frey filaments were processed to calculate an average force threshold for each animal. Every threshold value (3.22, 3.61 and 3.84) was multiplied by the number of recording sites displaying that threshold and the sum of these weighted values was divided by the total number of sites tested. A mean threshold was then calculated for each experimental group. Statistical treatment was done with ANOVA supplemented with multiple comparisons (Newman-Keuls post-hoc test) (Statistica, Statsoft). Statistical significance was set at P<0.05. Results are presented as mean ± standard deviation.

### Histological procedure

After the mapping session, the rats were given a lethal dose of pentobarbital (150 mg/kg, i.p., CEVA) and transcardially perfused with 0.9% physiological saline followed by a solution containing 4% paraformaldehyde in 0.1% sodium phosphate-buffered (pH: 7.4). Then, the brains were carefully dissected out and post-fixed for 12 h at 4°C in a 4% paraformaldehyde solution containing 10% sucrose in phosphate buffer. The blocks were cryoprotected by successive transfers into increasing concentrations (10%, 20% and 30%) of sucrose solution in 0.1 M phosphate buffer saline (PBS) for 72 h at 4°C. For histological examination, the brains were frozen using carbon dioxide and 40-µm-thick coronal sections centered on the cortical lesions were performed using a cryostat. Histological sections were mounted on slides, then stained with cresyl violet in order to assess morphological alterations occurring within the lesioned zone (Fig. 2 B).

## Results

The experimental data are based on high-density electrophysiological recordings obtained during double-mapping procedures in each of the 32 rats. On average, 123 and 118 cortical sites were recorded for individual mapping sessions before and after the lesion induction, respectively. In addition, an average of 32 recordings was used for the functional delimitation of the damaged area acutely assessed in each rat.

The percentage of tissue loss induced by the lesion was evaluated as a relative decrease in the reference polygon areas delineated using stable vascular landmarks on the images of the cortex surface (Fig. 3). On average, the loss of cortical tissue was not similar across all groups ([F(3,28) = 11.44; P<0.0001]. The tissue loss recorded in the NT1 rats (28.6±4.1%, SD) was similar to that in the NT2 (26.3±3.8%; P: n.s.) and was not different from the loss found in the DT animals (25.6±3.3%; P = n.s.). However, the tissue loss was significantly greater in these three groups than in the ET rats (20.0±2.7%; P<0.001).

**Figure 4:**
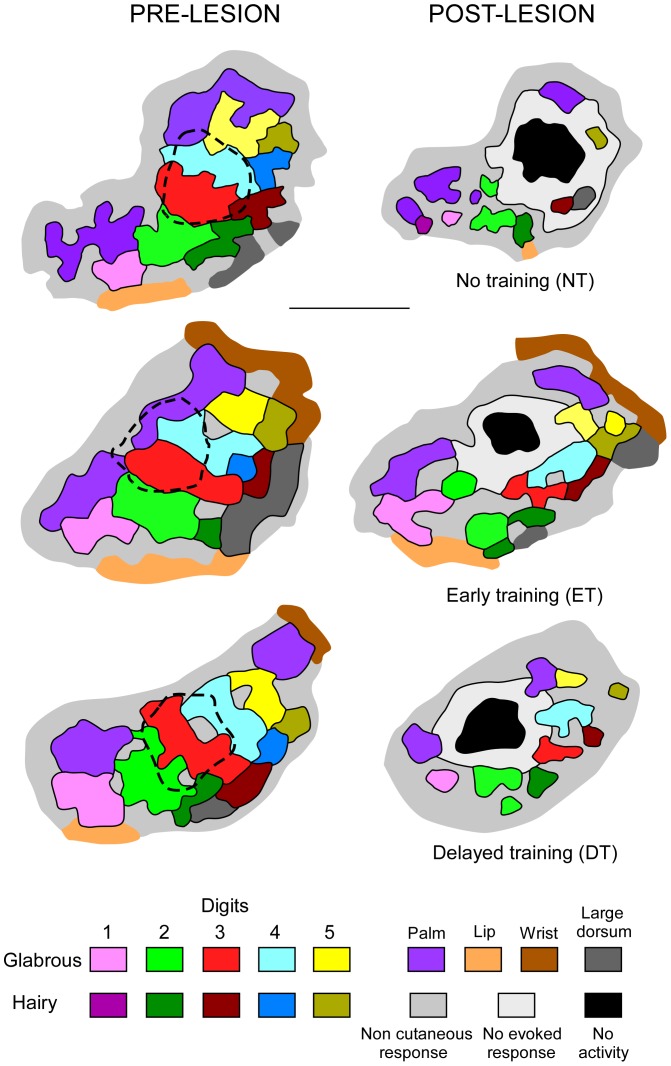
Forepaw representations obtained prior to and after the lesion induction. Examples of electrophysiological maps obtained in a rat which was not subjected to tactile stimulation (NT) and in rats subjected to tactile stimulation from the 3^rd^ to the 8^th^ days (early stimulation; ET) or from the 8^th^ to the 13^th^ days (delayed stimulation; DT) after the lesion induction, while cortical remapping was performed on the 9^th^ or 14^th^ days, respectively. The postlesion map in the NT rat was obtained on the 9^th^ day postlesion (NT1). For the sake of legibility, no map is shown for NT2 rats in which remapping was performed on the 14^th^ day postlesion, as NT1 and NT2 maps were very similar. The injured areas functionally defined on the basis of neuronal recordings are outlined on the pre-lesion maps (dashed lines). The cortical sectors electrophysiologically silent (black area) or displaying decreased spontaneous activity with no elicited response (light grey area) are illustrated on the post-lesion maps. Note that the best preservation of cutaneous representational zones was recorded in the ET rat, whereas the most degraded map was found in the NT rat.

As shown in previous studies [23], [24], the somatotopic organization of the forepaw representation within the intact S1 cortex of rats displays invariant organizational features despite inter-individual differences in representational details (Fig. 4). Briefly, the forepaw map is somatotopically organized in the rostrolateral-caudomedial direction, from the thenar eminence and digit 1 to the hypothenar eminence and digit 5, and in the rostromedial-caudolateral direction, from the palmar pads to the glabrous and hairy skin surfaces of digits. The cortical regions representing contiguous skin surfaces are usually interspersed with small noncutaneous islets. Cortical sites along the lateral margins of the cutaneous map either respond to stimulation of the chin or lower lip, to stimulation of non-cutaneous structures, or are unresponsive. The medial margin of the forepaw map is delineated by cortical sectors responding to strong taps on the palmar pads. The caudal edge of the map is delimited by cortical sites excited either by taps on the wrist or by gentle deflection of the long vibrissal hairs of the wrist.

### Effects of rehabilitative tactile training on remaining forepaw representational zones

As the number of task learning sessions preceding the first mapping varied among rats, we assessed whether the stimulation delivered during these sessions had an impact on the areal extent of forepaw representation. Comparison of the four experimental groups regarding the prelesion total area of cutaneous forepaw map (NT1: 1.68±0.20 mm^2^; NT2: 1.74±0.18 mm^2^; ET: 1.78±0.16 mm^2^; DT: 1.80±0.23 mm^2^) revealed no significant differences [F(3,28) = 0.87; P: n.s.]. Further analysis also showed that cortical zones serving the digit glabrous skin primarily engaged in the tactile stimulation procedure were not different between groups (NT1: 1.43±0.16 mm^2^; NT2: 1.41±0.15 mm^2^; ET: 1.53±0.14 mm^2^; DT: 1.49±0.18 mm^2^) [F(3,28) = 1.06; P: n.s.].

As previously described [20], the injured cortical area was characterized by a zone of neurons displaying strongly decreased spontaneous activity and no evoked responses that surrounded a completely silent core. The mean areas of the focal ischemic lesions functionally defined during the second hour postlesion were similar in the four groups of rats [NT1: 0.46±0.14 mm^2^; NT2: 0.52±0.11 mm^2^; ET: 0.51±0.13 mm^2^; DT: 0.54±0.16 mm^2^] [F(3,28) = 0.63; P: n.s.]. The forepaw maps obtained in the NT rats 9 or 14 days after the lesion induction showed an expansion of the initial representational loss, the silent cortical zone being surrounded by strongly depressed responses [30] and emerging non-cutaneous zones that created discontinuities in formerly contiguous sectors of cutaneous representations. This additional loss of cutaneous territories averaged −62.61±9.50% and −69.25±10.05% in the NT1 and NT2 rats, respectively. The further loss was significantly smaller in the rats subjected to tactile stimulation (ET: −21.04±8.45% and DT: −35.19±7.49%) [F(3,28) = 40.26; P<0.0001]. However, it was greater in the DT than in the ET animals (P<0.009) indicating a larger spared area of forepaw maps after early postlesion tactile stimulations (Fig. 4).

The ischemic lesion targeted the cortical zones serving the glabrous skin of digits 2-3-4 and part of the palm sectors of the forepaw representation. Post-lesion maps showed that the lost representational zones were not restored in the perilesion area. As training-induced cutaneous stimulation was primarily applied on the glabrous skin of digits, we attempted to determine whether the stimulation-dependent preservation had a different impact on the digit, palm and dorsum representational sectors of the forepaw map. ANOVA gave a significant effect of map region [F(2,84) = 7.38; P<0.001] and group [F(3,84) = 129.86; P<0.0001], and a significant region by group interaction F(6,84) = 17.45; P<0.0001]. The digit glabrous skin territories were found to be better preserved in the ET (−14.36±9.74%) than DT rats (−31.33±12.17%) (P<0.005). A greater expansion of the initial loss of digit representational was recorded in the NT1 (−71.20±11.84%) and NT2 rats (−75.17±8.72%; P<0.0001). The palm representation was also relatively well preserved in the ET (−34.55±5.39%) compared to the DT rats (−53.99±4.72%) (P<0.0001) in which the additional loss was similar to that recorded in the NT rats (NT1: −58.62±6.41%; NT2: −62.28±4.27%; P = 0.19 and P = 0.32, respectively) (Fig. 4). In both ET and DT rats, the preservation effect found for glabrous digit representations was generally more prominent than that recorded for the palm sectors (P<0.0001). We searched for a possible influence of tactile training on the cortical sectors serving the hairy skin, located laterally in the forepaw map. The loss of hairy skin representation was also smaller in the ET rats (−32.46±5.83%) (P<0.0001) than in the DT (−58.30±5.68%) and NT rats (NT1: −65.70±7.21%; NT2: −65.50±4.27%), although the beneficial effect of training in the ET animals was less pronounced than that found for the glabrous digit representation (P<0.0001).

### Neuronal receptive field size and responsiveness in spared regions of the forepaw map

We first checked whether the experimental groups were different regarding RF sizes recorded prior to the ischemic damage. As classically described in the literature, glabrous skin RFs located on the digit or palm skin surfaces displayed similar sizes, on average, whereas dorsal skin RFs were larger (see Table 1). In this respect, no differences among experimental groups were found [F(3,84) = 1.85; P = 0.14.]. Then, we searched for possible effects of tactile training on the neuronal RF sizes recorded in the spared cortical sectors of the postlesion maps. We first computed the mean RF sizes for the digit, palm and hairy skin RFs within each experimental group and separately for the pre- and postlesion conditions. Then, we averaged the corresponding post - prelesion differences in RF sizes. ANOVA yielded no main effects of group [F(3,84) = 2.44; P = 0.07], location on skin surfaces [F(2,84) = 2.21; P = 0.12] and no location by group interaction [F(6,84) = 1.01; P = 0.42]. Therefore, the neurons recorded within the spared cortical territories of the forepaw map displayed RF sizes which were not altered by the tactile training.

**Table 1:** Sizes of glabrous and hairy skin receptive fields recorded in trained and untrained rats.

GROUP	LOCATION	PRELESION			POSTLESION			(POST-PRE)/POST		
		Mean	SD	N	Mean	SD	N	Mean	SD	N
Early Training	digit	3.85	0.61	8	4.18	0.56	8	0.08	8	0.25
Early Training	pad	4.47	0.51	8	4.05	0.58	8	−0.10	8	0.13
Early Training	dorsal	10.80	1.69	8	10.12	1.24	8	−0.07	8	0.08
Delayed Training	digit	3.12	0.58	8	3.02	0.92	8	−0.03	8	0.12
Delayed Training	pad	4.00	0.75	8	4.10	0.53	8	0.02	8	0.29
Delayed Training	dorsal	9.52	1.13	8	9.40	0.98	8	0.02	8	0.11
No Training 1	digit	3.32	1.16	8	3.74	0.78	8	0.11	8	0.22
No Training 1	pad	4.85	0.91	8	5.41	0.88	8	0.10	8	0.35
No Training 1	dorsal	9.58	2.76	8	10.92	2.31	8	0.12	8	0.22
No Training 2	digit	3.51	0.94	8	3.68	0.84	8	0.04	8	0.18
No Training 2	pad	4.86	0.92	8	5.56	0.63	8	0.13	8	0.22
No Training 2	dorsal	10.46	2.16	8	11.47	2.36	8	0.09	8	0.13

Mean values (±SD) of ventral and dorsal skin receptive fields (RFs) recorded in the four experimental groups of rats before and after the lesion. The RFs are expressed in mm^2^. The mean values are distinguished according to RF location on glabrous surfaces of digits and pads, and on hairy dorsal skin surfaces. No effects of lesion or training were found when comparing experimental groups.

**Figure 5:**
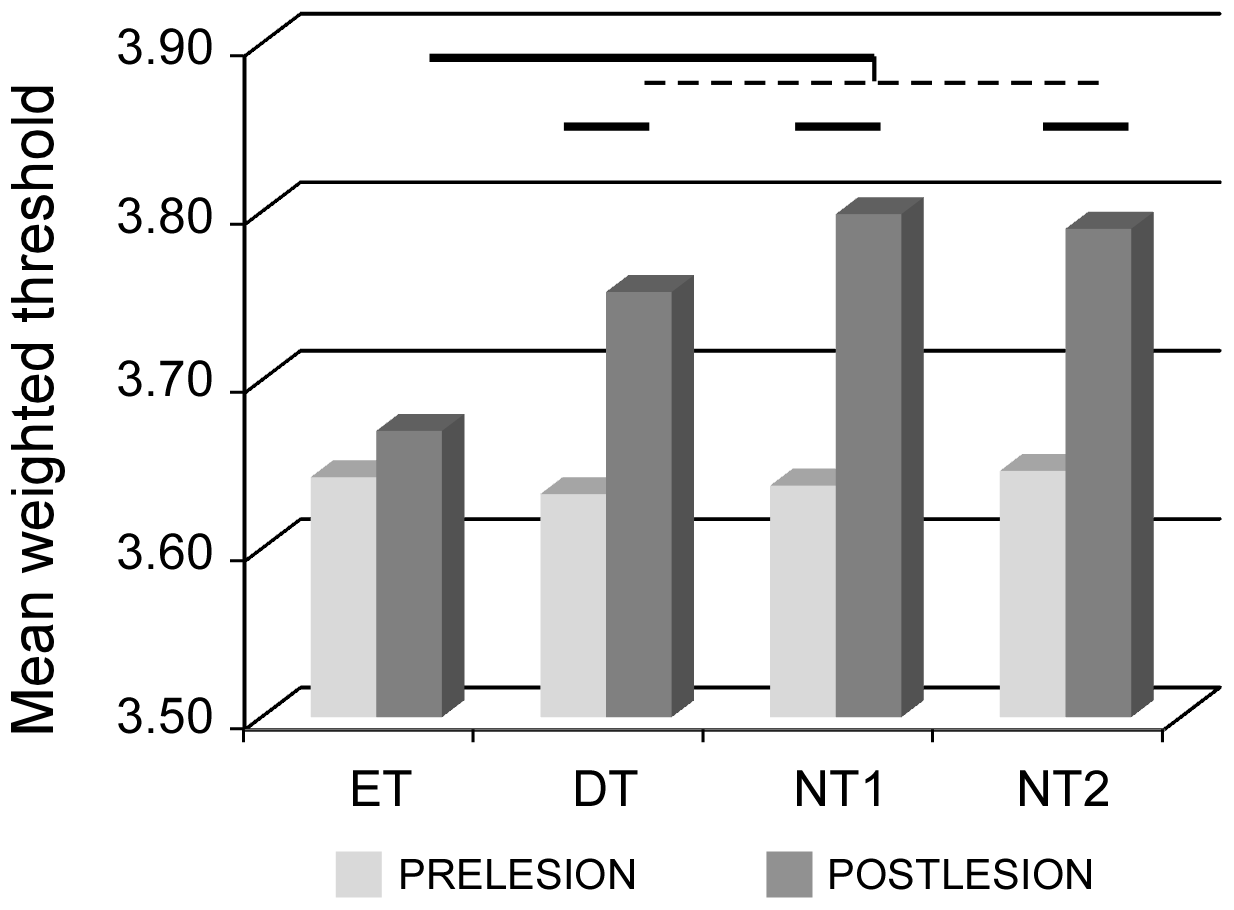
Postlesion changes in neuronal responsiveness. The histograms show mean weighted thresholds determined with force-calibrated Von Frey filaments applied at the centers of cutaneous RFs. Pre- versus post-lesion significant differences yielded by post-hoc tests are indicated by horizontal bars (P<0.0001). Postlesion values recorded in ET rats differed from those obtained in all other groups (DT, NT1 and NT2), whereas no differences were found between those three groups. Note that neuronal responsiveness was similar before and 9 days after the lesion in ET rats.

While determination of RF size provides information about the spatial grain of cortical representations, assessment of neuronal responsiveness provides insights into cortical neurons' excitability. We thus examined whether cortical excitability was influenced by postlesion tactile training. The weighted mean thresholds obtained using the force-calibrated Von Frey filaments applied at the centers of RFs on the glabrous skin of digits or palm were taken as indicative of the cortical responsiveness. As shown in Fig. 5, all experimental groups displayed, on average, very similar mechanical thresholds before the lesion [F(3,28) = 0.19; P = 0.90]. However, ANOVA yielded a main effect of group for the data collected after the lesion [F(3,28) = 21.18; P<0.002]. The force thresholds obtained in the ET rats were not altered after the lesion (P = 0.93), whereas those recorded during the postlesion mapping in the DT, NT1 and NT2 rats were higher than the corresponding prelesion thresholds, thus indicating an overall decrease in sensitivity to tactile stimulation in these three groups (Fig. 5).

## Discussion

### Early cutaneous stimulation exerts a neuroprotective effect by reducing tissue loss after focal ischemic damage

Our measurements based on stable vascular landmarks on the cortical surface indicate a substantial shrinkage that attests to a tissue loss centered on the core of the infarct. We found that this tissue loss was similar in the NT1 and NT2 rats, i.e. did not differ between 9 and 14 days after the lesion induction, respectively. This result corroborates previous studies using cortical compression or endothelin-1 to induce a restricted ischemic infarct to the S1 cortex which showed that cell death increase during the early postlesion days, is at a maximum at 3–5 days and does not evolve over the subsequent days and weeks [31], [32], [33], [34].

In previous studies, we showed that after ischemia targeting the forelimb area of S1, housing rats as early as 24 hours after the lesion in enriched environments (EE) promoting sensorimotor experience did not impact on tissue loss and gliosis volumes measured on histological sections [33] or cortical surface images [20]. These findings corroborated earlier studies on the effects of housing conditions after extensive cortical damage induced by MCAO [35]. By contrast, lesion volume induced by traumatic brain injury was found to be smaller in rats recovering in EE [36], [37]. However, in all these studies, physical activity and social interactions of the rats in the EE were not assessed, thus casting doubt on whether the animals benefited from the EE during the first days postlesion. In contrast, in our study the animals were actively engaged in a challenging rewarded task.

Cortical activation has been shown to exert a key influence on the extent of infarct through a gradual reperfusion of the ischemic penumbra, a region of moderate blood flow surrounding the core ischemic zone that eventually infarcts. Functional stimulation is accompanied by an increase in regional cerebral blood flow (CBF) in activated brain regions. Even when CBF is reduced by as much as 90% after temporary MCAO, forepaw stimulation is still able to elicit an increase in blood flow in the somatosensory cortex [38]. This finding is consistent with the observation that electrical forepaw stimulation during reversible focal ischemia reduces cortical infarct volume [39]. Furthermore, single whisker stimulation delivered within a one or two hour time-window following onset of MCAO was found to completely protect the cortex from impending stroke damage, to restore cortical function and resulted in full preservation of sensorimotor capabilities. Using blood flow imaging, these authors provided evidence that this sensory stimulus-induced reperfusion was due to reversed blood flow into MCA branches from an alternate arterial source via collateral arteries anastomosing with branches of the occluded vascular tree. However, when initiated three hours after the lesion, whisker stimulation not only failed to fully protect the cortex but even resulted in larger infarct than when stimulation was not delivered [8], [ 40]; [ for review see 41]. These findings are pertinent to the observation that pharmacologically induced reperfusion of peri-infarcted brain tissue within the first few hours after focal ischemia preserves perilesion cortical representations that otherwise start to be deteriorated [19]. Collectively, these studies underscore a very early time window for the protective effects of sensory stimulation known to induce an increase in regional cerebral blood flow mediating cellular metabolic normalization. Sensory stimulation had deleterious effects when administered after the first three hours in Frostig's studies, when cortical tissue is particularly vulnerable to experience-dependent neuronal excitability and exacerbation of damage. However, it is noteworthy that a pattern of delayed ischemic damage appears after 3 days following a mild reversible ischemic injury and is completed at about 1 week post-occlusion, presumably as a result of apoptosis or secondary energy failure due to DNA repair [42], [43]. It is well established that cell death in the ischemic core is triggered by necrosis whereas that occurring in the penumbra is predominately mediated by apoptosis [44]. Moreover, apoptosis being a reversible process, therapeutic interventions have the potential to prevent or limit cell death within a late stage of cerebral ischemia [45].

Previous studies showed that early training of the impaired limb after stroke can have either a beneficial or deleterious impact. Early initiation of a forced use of the impaired limb by restricting the unimpaired forelimb [9], [11], or early training of the affected forelimb [46] immediately after focal ischemia was shown to increase lesion volume and impede sensorimotor recovery of the affected limb in the rat. Conversely, constraining the intact limb [10] or training the affected limb [46] seven days after the onset of brain damage did not induce enlarged cortical infarct volumes and resulted in better limb use. Taken together, these studies and those by Frostig et al. previously mentioned [8], [40], raised the issue of an early vulnerable period for neurons in the peri-infarct cortical networks resulting from use-dependent exacerbation of cellular events involving changes in glutamate and GABA levels (see below). Our data show a greater preservation of cortical tissue integrity in the ET rats than in the NT or DT animals. This result indicates that moderate, short-duration tactile stimulation inducing selective activation of S1 peri-infarct zone during the first week postlesion has a neuroprotective potential. This finding is in concordance with a study showing that acrobatic training started 2 days after lesion to the somatosensory and motor hindlimb representations limited the loss of cortical tissue in the perilesion area [47]. This training which lasted about 30 min per day was gradually increased in intensity during the first week. In this study, no tissue preservation was found in rats receiving simple exercise in a straight runway. The time-limited window which corresponds to the period of delayed ischemic injury may be considered as critical for early onset of use-dependent neuronal changes in the area of potentially viable cells surrounding an ischemic core. This notion is consistent with the fact that early irreversible changes occur in the ischemic core, whereas the ischemic penumbra that surrounds this core and receives more collateral blood flow suffers milder and delayed damage. The perilesion area can thus escape programmed cellular death, benefit from reperfusion and neuroprotective strategies [48], [49]. Cortical tissue preservation may be partially attributed to upregulation of neurotrophins, proteins involved in neuronal survival and in activity-dependent plasticity, as revealed by the powerful modulation of BDNF by increased physical exercise [50] learning [51] and EE [52]. With regard to our results, even though cortical tissue loss was greater in DT than in ET rats, it cannot be ruled out that delayed stimulation may also exert protective effects if properly adjusted in intensity, as indicated by the observation that EE combined with multimodal stimulation applied for 7–15 days after traumatic brain injury substantially reduced lesion volume [53].

### Cutaneous stimulation limits the secondary loss of representation in the preserved perilesion area following focal cortical ischemia

The present study complements earlier findings showing that focal ischemic injury expands rapidly, thus resulting in an additional loss of representations, but also causes deterioration in the organizational features in the spared perilesion sectors of cortical maps [19]. It thus appears that early intervention is needed to counteract the halo of electrophysiological disturbances in the neuronal network surrounding the ischemic core. Defect in neuronal rescue and survival, together with enduring map alterations after restricted ischemic infarct to the forepaw area were found to cause conspicuous impairments in the animals' ability to produce fine motor adjustments regulated by somatosensory inputs [54].

A major observation in the present study is that mild cutaneous stimulation initiated during the first week postlesion limits the secondary map degradation in the peri-infarct zone. We have previously shown that after a focal ischemic lesion to the forepaw area in S1, rats exposed to an impoverished environment for three weeks exhibited an enlargement of the ischemic zone as well as a compression and fragmentation of the remaining cutaneous forepaw representation within the spared cortical sectors surrounding the lesion. In contrast, in animals housed in enriched conditions, the ischemic zone did not grow and only a limited deterioration of the forepaw map was found in the peri-infarct zone, with a preservation of most representational sectors [20]. These findings indicate that a substantial amount of the cortical tissue in the perilesion cortex can escape the infarction process and display normal neurophysiological responses. The present mapping data show that mild cutaneous stimulation of the forepaw, even for short daily periods during the first postlesion week, was sufficient to induce a preservation effect similar to that reported in the rats exposed to EE for 3 weeks. The postlesion additional loss in cutaneous representation was limited to about 20% of the acute forepaw map area, when cutaneous stimulation was initiated early. An additional loss of about 60% was recorded in non-stimulated animals at the 9^th^ and 14^th^ days postlesion. Stimulation delayed to the second week postlesion limited the supplementary loss to about 35%, thus indicating a lesser beneficial effect than that of early stimulation. In accordance with our results showing a substantial impact of short-duration practice, cortical motor output to the paretic muscles in stroke patients was found to be significantly expanded and manual dexterity was transiently improved after a single session of constraint-induced movement therapy [55].

### Are the protective effects of sensory stimulation specific for the stimulated skin surfaces?

Interestingly, the expanded loss of representation recorded for the glabrous representation of digits after early rehabilitative practice was about twice smaller than the loss observed for palm or dorsal skin surface representations, despite the fact that the ischemic injury primarily targeted the glabrous digit area and part of the palm sectors of the maps. The beneficial influence of delayed stimulation on the ventral digit representation was still significant, although less pronounced than that of early stimulation. By contrast, the delayed stimulation did not impact on the palm and dorsum areas of the forepaw map. Therefore, the neuroprotective effect of cutaneous stimulation was more conspicuous on the peri-infarct cortical sectors serving the stimulated skin surfaces and was most effective during the first postlesion week.

In line with those findings, Nudo et al. [56] reported that following focal ischemic damage to M1 in monkeys that benefited from manual dexterity training of the impaired forelimb from the 5^th^ day postlesion, the undamaged sectors of the hand motor map were preserved, unlike in untrained animals, suggesting that the efficacy of local network neurons projecting to hand motoneurons was maintained in these trained monkeys. It is of relevance to the present study that when the animals experienced delayed rehabilitative training one month after the lesion, the lost movement representations were not recovered [57]. Using the same rehabilitative manual dexterity training as in Nudo's study, but after a focal ischemic lesion that targeted the cutaneous representation of two digits in area 3b of S1 and impaired digital dexterity primarily involving these digits, we found that new representations emerged several millimeters away from the lesion, in the cortical sector of areas 1 and 3a adjacent to area 3b, several months after the lesion induction [58]. The pattern of cortical changes across monkeys was found to be idiosyncratic, depending on individual digit use and retrieval strategies. The skin surfaces that regained a representation in ectopic cortical regions were located on the fingers crucially engaged in the retrieval task during the rehabilitative training period initiated within 3–14 days after the lesion induction.

Nevertheless, as local activation of cortical networks spreads into neighboring cortical regions via an underlying network of long-range connections [59], sensory stimulation exhibits protective effects over larger cortical zones than that defined by anatomical thalamo-cortical projections [8]. Consistently with this notion, our results show that early stimulation had a beneficial effect, although less pronounced, on peri-infarct cortical sectors serving non-stimulated skin surfaces. It is worth mentioning, however, that delayed stimulation strongly attenuated this diffuse protective effect (see also below).

### Experience-dependent protective effects on perilesion tissue: preservation or restoration of cortical map organization?

It is of interest to determine whether the stimulation-induced functional neuroprotection reported herein reflects a sparing of cortical territories beyond the initial lesion boundaries or a restoration of transiently lost representations. As the ischemic damage gradually expanded over the first few days postlesion, we assume that early sensory stimulation counteracted the propagation of ischemic damage and promoted metabolic normalization and survival of neurons in the peri-infarct cortex. As a result, the electrophysiological response properties of the constituent neurons were preserved and thereby somatotopic organization was maintained in the area surrounding the lesion.

We found that the overall representational loss was similar in both groups of non-stimulated rats in which the forepaw area was remapped on the 9^th^ or 14^th^ days postlesion. This result corroborates the stabilization of the lesion expansion at the end of the first week. One cannot exclude that somatosensory stimulation generated by the animals' behavior in their standard housing environment was sufficient to promote tissue reperfusion by collateral vessels, thereby limiting propagation of the focal ischemic damage beyond the end of the first week postlesion. Nevertheless, restoration of cutaneous representation did not occur spontaneously over the second week postlesion. Thus, at the time when delayed cutaneous stimulation was initiated, i.e. on the 8th day, the degradation of forepaw representation was established. Our data show that the extent of the remaining forepaw area was greater in the rats with delayed stimulation than in the non-trained rats whose cortex was remapped at the same postlesion time, i.e. on the 14^th^ day. It is very likely that the representational loss observed in the non-trained rats at the end of the first week postlesion had similarly affected the rats subjected to delayed training. In the latter, however, the representational loss had been partially compensated for over the second week, as a result of the cutaneous stimulation delivered during this period. The hypothesis of restoration guided by sensory experience is supported by the finding that the cortical zones serving the glabrous skin of digits were partly reestablished in the rats trained from the 8^th^ day, in contrast to palm and dorsum areas which remained similarly degraded in these rats and non-stimulated congeners. This hypothesis implies that the delayed stimulation was efficient enough to rescue some neuronal populations within a rim of tissue surrounding the ischemic core and allowed subsequent reinstatement of topographically organized cutaneous representations within this salvageable tissue. It is thus likely that neurons in this “penumbral” zone, had been temporarily depressed, but then regained some responsiveness under the influence of experience-driven reactivation of cortical neuronal circuitry.

To sum up, early mild training was found to limit expansion of the ischemic lesion and substantially maintain the neuronal responsiveness to tactile stimulation, thereby preserving somatotopic map arrangement in the peri-infarct cortical cortex. By contrast, delayed training only partially restored lost representations in a use-dependent manner within the spared peri-infarct cortical area.

### Receptive field sizes and responsiveness are preserved in the perilesion cortex following early tactile stimulation

Cortical maps are dynamically maintained or reshaped through use-dependent ongoing adjustments in the balance of excitatory and inhibitory neurotransmission modifying RF location, size and sensitivity. After stroke, this fine-tuned balance is compromised in the peri-infarct cortex by various changes in cortical signalling including GABA_A_ receptor subunits, GABA transporters or glutamate receptor expression.

Our data indicate that in all groups of rats, except those benefiting from early sensory stimulation, cortical sites in the spared cortical territories of the forepaw map exhibited a significant reduction in neuronal excitability, as assessed by higher mechanical thresholds of response to cutaneous stimulation at the centers of RFs, i.e. their most sensitive areas where maximum excitation and inhibition coincide [60], [61]. The diminished neuronal responsiveness to cutaneous inputs very likely contributed to the overall reduction of cutaneous representation due to substantial loss of suprathreshold responses. We have previously reported a diminution in neuronal responsiveness to light tactile stimulation at 3 weeks post-injury in the spared cortical zones surrounding a focal ischemic infarct [20]. Preservation of RF sensitivity found only in the early-trained rats suggests that somatosensory inputs contributed to maintain cortical excitability and presumably a better balance between inhibitory and excitatory transmission within the peri-infarct cortex.

Previous studies which assessed GABA_A_ synaptic subunit receptors reported that phasic GABA inhibition, which leads to rapid and transient hyperpolarization, is reduced in the peri-infarct cortex, mostly at the end of the first week after stroke [62], [63], [64], [65], [66], [67]. In contrast to phasic GABA inhibition, tonic or extrasynaptic GABA signaling which controls the overall membrane potential of postsynaptic neurons and their propensity to fire in response to excitatory inputs [68, for review] has been shown to increase in peri-infarct cortex, probably as a result of diminished GABA uptake in reactive astrocytes [69], [70]. Interestingly, administration of GABA_A_ receptor alpha5 inverse agonists was found to reverse the poststroke increase in tonic GABA current and restore cellular excitability in pyramidal neurons [71]. Moreover, this treatment improved motor recovery when given chronically, starting 3 days after stroke in mice. The available evidence suggests that phasic GABA signalling is reduced, whereas tonic GABA signalling is enhanced during the first weeks after focal stroke. It has been argued that the overall effect of decreased phasic inhibition and increased tonic inhibition is a diminished neuronal excitability which, when reversed, promotes functional remapping and behavioural recovery [see 72 and 73, for reviews]. Therefore, it is very plausible that alteration in tonic GABA inhibition leading to cortical neurons' hypoexcitability contributed to the reduced responsiveness reported in the present study. In fact, the diminished neuronal responsiveness recorded in the perilesion cortical sectors in NT and DT rats is difficult to reconcile with an overall decreased inhibition. In addition, one might have expected that such a lesion-induced reduction of inhibition resulted in an enlargement of cutaneous RFs by unmasking previously subthreshold inputs [74], [75], [76]. No decrease in RF responsiveness was recorded in the early-trained rats, indicating that somatosensory stimulation tended to maintain cortical neuron excitability, presumably through a preserved excitatory/inhibitory balance in most sectors of the remaining map, as proposed above to explain the lack of change in RF size. In congruence with the fact that GABA_A_ receptor agonists given at the time of stroke decrease the infarct volume by counteracting the harmful effects of the glutamate-induced excitotoxic cascade [77], dampening tonic inhibition from stroke onset resulted in increased cell death [71]. Therefore, increase in tonic GABA inhibition during the acute phase of stroke seems to act as a neuroprotective mechanism, whereas tonic GABA inhibition during the subacute phase (first weeks) would curtail neuronal plasticity in the peri-infarct zone. It seems likely that enduring increase in tonic GABA inhibition prevents sensory experience from remodeling cortical networks during a critical time window, thereby hindering recovery. Interestingly, the time of inflection from protective to deleterious effects of increased tonic GABA signaling was found to occur as early as 3 days poststroke in mice [71].

Glutamate signaling also plays a dual role in the fate of neurons after stroke. It is well established that excessive glutamate release during acute phase of ischemia has an excitotoxic effect on peri-infarct tissue. This effect is likely to mediate the deleterious influence of early intensive training on infarct volume and recovery already mentioned in the present paper. Studies addressing cortical reorganization in the surrounding area of focal ischemic damage to sensorimotor cortex have reported an increased glutamatergic response mediated by the NMDA receptors [63], [64]; [66]. Increase in binding density of NMDA receptors was found to appear after a delay of 14 days postlesion in forelimb area of the motor cortex and later in the somatosensory cortex [66]. However, immunoblotting analysis of receptor proteins showed that in the center and immediate surrounding area of an ischemic cortical injury, NMDAR1 subunit was strongly reduced during the subacute phase of ischemia, i.e. the first 2 weeks postlesion, the lowest levels in NR1 coinciding in time with the largest functionally silent region, i.e. during the first week [78]. Similarly, glutamate levels were greatly diminished during the first 2 weeks postlesion. Interestingly, a gradual increase in NR1 and glutamate levels was found to develop from the end of the second week to the 5^th^ week, at a time when functional reorganization was observed [67]. The reduced glutamate neurotransmission during the first 2 weeks postlesion is consistent with the loss of cutaneous representation in the perilesion area recorded in the NT rats. One can assume that this reduction in glutamate neurotransmission may have been counteracted by the early training favoring preservation of perilesion representational zones in the ET rats and partial restoration of lost representations in the DT rats. It is noteworthy that experimentally-induced increase in glutamatergic excitability in the subacute phase of stroke was found to parallel recovery. For example, while early administration of AMPA receptor agonist worsened cell damage, when this treatment was given after a 5-day delay from stroke, infarct size was not modified, but limb motor control improved in a dose-dependent way suggesting a beneficial effect on recovering tissue [79]. Furthermore, blocking AMPA receptor signaling during 5 days after the lesion induction impaired motor control. Interestingly, this study also provides evidence that BDNF release in the peri-infarct cortex, and not in other brain regions, mediated the recovery effect of enhancing AMPA receptor signaling. On the basis of this finding, Carmichael [72] proposed that the recovering circuitry in peri-infarct cortex may be primed for activity-dependent BDNF release. It is of interest that BDNF application on NMDA receptor subunits increases NR1 and NR2A protein levels, decreases NR2B levels, with associated effects including enhanced calcium response to NMDA activation, increased necrosis and reduced apoptosis [80]. Furthermore, BDNF is known to regulate synaptic transmission and activity-dependent plasticity [81], [82], to promote survival and sprouting of dendrites and axons [83], [84], as well as synaptogenesis [85] and angiogenesis [86]. Interestingly, disrupting the expression of BDNF after focal stroke prevented training-induced recovery of skilled reaching in rats [87].

The available evidence from the literature shows that both temporal window and intensity of practice are crucial to promote neuroprotective and optimize neuroplasticity mechanisms operating in the peri-infarct cortex, as the influence of activity-dependent neurotransmission can be beneficial or detrimental depending on timing and intensity of rehabilitative procedures. In the present study, the stability of both RF size and responsiveness recorded in the early-trained rats strongly suggests that the balance of excitatory and inhibitory influences within vulnerable neural networks of the perilesion cortex was relatively well maintained as a result of early and mild cutaneous stimulation. The finding that the rats subjected to delayed training exhibited unchanged RF sizes, but decreased excitability in the restored cortical sectors, is consistent with incomplete re-establishment of the excitatory/inhibitory balance in the corresponding neuronal networks.

Together, our results show that moderate, but focused and challenging rehabilitative practice during an early critical period after small ischemic injury promotes protective mechanisms limiting tissue loss and sparing cortical neural representations within the peri-infarct cortex. This sparing effect which occurs very likely through local reperfusion, metabolic normalization and preservation of the balance of excitation and inhibition in the perilesion cortical networks declines with time. Under our experimental conditions, delaying this mild practice by one week was found to impede the protective effect of early sensory stimulation, as lesion expansion was not optimally counteracted. Nevertheless, experience-dependent plasticity was found to promote partial restoration of lost representations in salvageable perilesion tissue. The experience-induced protective effect of immediate or early practice may so far have been underestimated, while the main focus has been on neuroprotective agents preventing or reducing secondary brain damage. Delaying the “window of opportunity” in rehabilitative practice may be effective in promoting cortical map reorganization, whereas it may not be the optimal strategy for use-dependent neuroprotection. It may be proposed that preservation or reinstatement of neurophysiological properties in peri-infarct cortex, through early and moderate practice, reduces functional deficits and thus promotes faster and more complete behavioral recovery. Properly designed early intervention aimed at preserving the peri-infarct tissue may prepare for later adaptive experience-dependent remapping through massed practice, constraint-induced therapy or TMS.
